# The histone deacetylase inhibitor M344 as a multifaceted therapy for pancreatic cancer

**DOI:** 10.1371/journal.pone.0273518

**Published:** 2022-09-20

**Authors:** Shelby M. Knoche, Gabrielle L. Brumfield, Benjamin T. Goetz, Bailee H. Sliker, Alaina C. Larson, Madeline T. Olson, Brittany J. Poelaert, Audrey Bavari, Ying Yan, Jennifer D. Black, Joyce C. Solheim

**Affiliations:** 1 Eppley Institute for Research in Cancer & Allied Diseases, Fred & Pamela Buffett Cancer Center, University of Nebraska Medical Center, Omaha, Nebraska, United States of America; 2 Department of Pharmaceutical Sciences, Fred & Pamela Buffett Cancer Center, University of Nebraska Medical Center, Omaha, Nebraska, United States of America; 3 University of Nebraska at Omaha, Omaha, NE, United States of America; 4 Department of Radiation Oncology, Fred & Pamela Buffett Cancer Center, University of Nebraska Medical Center, Omaha, Nebraska, United States of America; University of South Alabama, UNITED STATES

## Abstract

The histone deacetylase (HDAC) inhibitor vorinostat, used with gemcitabine and other therapies, has been effective in treatment of experimental models of pancreatic cancer. In this study, we demonstrated that M344, an HDAC inhibitor, is efficacious against pancreatic cancer *in vitro* and *in vivo*, alone or with gemcitabine. By 24 hours post-treatment, M344 augments the population of pancreatic cancer cells in G_1_, and at a later time point (48 hours) it increases apoptosis. M344 inhibits histone H3 deacetylation and slows pancreatic cancer cell proliferation better than vorinostat, and it does not decrease the viability of a non-malignant cell line more than vorinostat. M344 also elevates pancreatic cancer cell major histocompatibility complex (MHC) class I molecule expression, potentially increasing the susceptibility of pancreatic cancer cells to T cell lysis. Taken together, our findings support further investigation of M344 as a pancreatic cancer treatment.

## Introduction

For pancreatic cancer, the currently approved treatments have had very little impact on survival [1]. In the effort to find new therapies, research by several groups has supported inhibitors of histone deacetylases (HDACs) as potential therapies for pancreatic cancer, as well as for other types of cancer [2–14]. HDACs remove acetyl moieties from lysine residues located on histones and, by causing changes in gene expression, HDAC inhibitors can reduce cancer cell growth [15–17]. For example, in a study on pancreatic cancer cell lines, the HDAC inhibitor vorinostat (also known as suberoylanilide hydroxamic acid, or SAHA) had an additive effect of decreasing growth when tested together with gemcitabine [5]. Vorinostat is now in clinical trials for pancreatic cancer treatment, and it is already FDA approved for cutaneous T cell lymphoma [11].

In addition, some HDAC inhibitors have been found to boost the expression of receptors on cancer cells that are targets for immune cells (such as cytotoxic T lymphocytes or natural killer cells) [18–27], thereby rendering the cancer cells more liable to destruction. For example, inhibition of HDACs has been demonstrated to raise the expression of major histocompatibility complex (MHC) class I molecules on glioma cells and increase the lysis of these cells by cytotoxic T lymphocytes [27]. In a clinical case report, patients whose Merkel cell carcinomas showed resistance to programmed cell death protein 1 (PD-1)/programmed death-ligand 1 (PD-L1) blockade and were subsequently treated with both an HDAC inhibitor and an immune checkpoint inhibitor had increased intratumoral cytotoxic T cell infiltration following the combination therapy [26].

The HDAC inhibitor 4-dimethylamino-N-(6-hydroxycarbamoylhexyl)-benzamide (also known as M344 [28]), like vorinostat, is effective at submicromolar concentrations for inhibiting HDACs 1, 2, 3, and 6 [11, 29]. M344 has been found to demonstrate anti-cancer activity against medulloblastoma, neuroblastoma, and rhabdoid tumor cell lines [30]. Furthermore, M344 facilitated the therapeutic response to radiation treatment in a squamous carcinoma cell model, and it has also been shown to have an anti-proliferative effect on breast cancer cells [31, 32].

In the current study, we demonstrated that M344 treatment of pancreatic cancer cells significantly reduces their proliferation and viability and greatly diminishes their ability to migrate. In comparison with vorinostat (using equimolar concentrations) we discovered that M344 was more effective at reducing pancreatic cancer cell proliferation. In addition, we showed that M344 in combination with gemcitabine, a chemotherapeutic approved for pancreatic cancer treatment, was superior to either M344 or gemcitabine alone at decreasing the proliferation and viability of pancreatic cancer cells. In an orthotopic xenograft mouse model of pancreatic cancer, M344 alone, or together with gemcitabine, significantly reduced tumor growth. We also found that M344 treatment results in augmentation of the expression of MHC class I molecules by pancreatic cancer cells, potentially increasing M344’s therapeutic effects by facilitating T cell killing of the tumor cells. In total, our work indicates that M344 strongly warrants further investigation as a novel pancreatic cancer therapy.

## Materials and methods

### Cell lines and culture media

The pancreatic cancer cell line S2-013 is a sub-clone of the SUIT-2 cell line, which was originally generated from a human pancreatic cancer metastasis to the liver [33–35]. The BxPC-3 and MIA PaCa-2 cell lines were derived from human primary pancreatic tumors [36–39], the T3M-4 pancreatic cancer cell line was generated from pancreatic cancer cells found in a lymph node [40, 41], and the CFPAC-1 cell line was produced from a human pancreatic cancer metastasis [42, 43]. The S2-013, BxPC-3, T3M-4, and CFPAC-1 cell lines were gifts from Dr. Michael A. (Tony) Hollingsworth (University of Nebraska Medical Center [UNMC], Omaha, NE USA) and were authenticated at the UNMC Molecular Diagnostics Facility by short tandem repeat deoxyribonucleic acid profiling analysis. The MIA PaCa-2 cell line was purchased from the American Type Culture Collection (Manassas, VA, USA). The HEK293 human embryonic kidney cell line [44] was donated by Dr. Amarnath Natarajan (UNMC).

The S2-013 and BxPC-3 cells were grown in RPMI 1640 (Life Technologies/Thermo Fisher Scientific, Waltham, MA, USA) supplemented with 10% fetal bovine serum (Atlantic Biologicals, Miami, FL, USA) that had been heat inactivated for 30 minutes at 56°C. The medum also contained 10 mM HEPES, 2 mM L-glutamine, 1X non-essential amino acids, 1 mM sodium pyruvate, 100 units/ml penicillin and 100 μg/mL streptomycin (all from stocks purchased from Thermo Fisher Scientific). The MIA PaCa-2, T3M-4, CFPAC-1, and HEK293 cells were grown in DMEM (Life Technologies/Thermo Fisher Scientific) containing the same supplements at identical concentrations as described above for the RPMI 1640 medium. The pancreatic cancer cell lines were demonstrated to be free of mycoplasma by testing with the MycoAlert Mycoplasma Detection Kit (Lonza, Basel, Switzerland).

### Antibodies and drugs

For the detection of human MHC class I molecules (i.e., human leukocyte antigen [HLA] class I molecules) by flow cytometry, the BB7.2 antibody (which recognizes peptide-occupied HLA-A2) [45, 46] and the HLA-B/C antibody (Clone B1.23.2, Thermo Fisher Scientific) were used. The B1.23.2 antibody recognizes HLA-B/C heavy chains that are associated with beta 2-microglobulin (and therefore also likely associated with peptide), but it also can recognize free HLA class I heavy chains [47]. The HC10 antibody (which recognizes peptide- and beta 2-microglobulin-free HLA-B and -C) was used for immunoblotting [48, 49]. The HC10 and BB7.2 antibodies were produced from hybridomas donated by Dr. Ted Hansen (Washington University, St. Louis, MO, USA). The anti-HSC70 antibody used as a control was obtained from Enzo Life Sciences (Farmingdale, NY, USA). The M344 (#A4105) and vorinostat (#A4084) utilized in experiments were purchased from APExBIO. The gemcitabine (#122111-03-9) for *in vitro* experiments was acquired from MilliporeSigma (Darmstadt, Germany), and the gemcitabine for *in vivo* experiments was obtained from the Nebraska Medicine Pharmacy Warehouse.

### Transwell migration assay

The cells were plated at 2 x 10^5^ cells/well on the day before treatment. On the following day, the cells were treated for 24 hours with 1 μM or 10 μM M344 in the experimental wells and with a corresponding volume of 0.1% dimethyl sulfoxide (DMSO; D12345, Thermo Fisher Scientific) in the control wells. Following this incubation, the cells were re-plated in 8-micron inserts at a concentration of 1x10^5^ cells in base maintenance medium (without fetal bovine serum but in the presence of 1 μM M344 or 10 μM M344 or 0.1% DMSO), and the cells were allowed to move to high fetal bovine serum (15%)-containing base maintenance medium for 24 hours. The inserts were then washed 3 times with cold phosphate-buffered saline (PBS), stained using the HEMA 3^TM^ STATpack (Thermo Fisher Scientific), and placed onto slides. Three random fields of each insert were photographed and counted to obtain the average number of migrating cells.

### Assays for cell proliferation and viability

Cell proliferation was analyzed by the MTT assay. The cells were plated at uniformly low density in 50 μl volumes in 96-well plates for the MTT assay, and the plates were incubated at 37°C for 24 hours. (The plating density was 2,500 cells/μL for the BxPC-3, T3M-4, and CFPAC-1 cell lines, 2,250 cells/100 μL for the S2-013 cell line, and 10,000 cells/100 μL for the MIA PaCa-2 cell line. A larger cell number was used for the MIA PaCa-2 cell line because it has a longer doubling time.) Media aliquots (50 μl volumes) containing the relevant drugs were added to the wells to produce the final treatment concentrations desired. At various time points after the start of treatment, a 50 μl volume of MTT reagent (thiazolyl blue tetrazolium bromide, 98%; L11939, Alfa Aesar/Thermo Fisher Scientific) was added to the 100 μl of medium in each well, and the plates were incubated at 37°C for 2 hours. Post-incubation, all liquid was removed from each well, 100 μl aliquots of DMSO were added to the wells, and the plates were read at 570 nm in a SpectraMax M5e or BioTek 800 TS Absorbance Reader.

Viability was monitored by suspending the cells in trypan blue stain and counting the cells on a hemocytometer. Live and dead cells were counted separately. The percentage of live cells was determined by dividing the number of live cells by the total number of cells (both live and dead) and multiplying by 100.

### Cell cycle analysis

To examine the impact of M344 treatment on the pancreatic cancer cell cycle, S2-013 cells were treated with 1 or 10 μM M344 (or 0.1% DMSO vehicle control) for 24, 48, or 72 hours. The cells (1.5 x 10^6^ per each treatment and time point) were washed in PBS, fixed with 80% ethanol for 1 hour, permeabilized with 0.1% NP-40 for 10 minutes, treated with Ribonuclease A (R4875, Sigma) in 0.1% NP-40 for 10 minutes, and stained with 0.416 mg/mL fluorescent dye propidium iodide (P-4170, Sigma) in 0.1% NP-40 for 30 minutes. The DNA content of a minimum of 20,000 cells was assessed by flow cytometry with a BD^TM^ LSR II flow cytometer (BD Bioscience, San Jose, CA) at the University of Nebraska Medical Center Flow Cytometry Facility. FlowJo^TM^ v10.7 Software (Ashland, OR, USA) was used for the data analysis.

### Caspase-3/7 activation assay

For assessment of changes in activated caspase-3 and -7 levels, 5 x 10^5^ S2-013 cells were plated in 10-cm cell culture dishes. After 24 hours, the cells were treated with 0.1% DMSO, 1 μM M344, or 10 μM M344 for 24, 48, or 72 hours. At each time point, the cells were trypsinized and 1 x 10^6^ cells per treatment were collected and resuspended. The cells were centrifuged for 5 minutes at 1,500 rpm in an Eppendorf 5810R centrifuge. The medium was removed by aspiration and the cells were resuspended in PBS containing 2% bovine serum albumin at 1 x 10^6^/3 ml, and 1 ml of each sample was aliquoted to 5-ml round-bottom polystyrene tubes (for a final concentration of 333,333 cells/ml/tube). The cells were stained using the Invitrogen CellEvent^TM^ Caspase 3/7 Green Flow Cytometry Assay Kit (#C10427 Thermo Fisher Scientific). Every sample received 1 μl of CellEvent^TM^ Caspase-3/7 Green Detection Reagent, which permeates cell membranes and has a nucleic acid-binding dye conjugated to a caspase 3/7 recognition sequence that, when cleaved, results in fluorescence. After gentle mixing, the tubes were incubated in the dark at room temperature for 1 hour. At 5 minutes before the end of the 1-hour incubation, 1 μL of SYTOX^TM^ AADvanced^TM^ Dead Cell Stain was added to each sample with gentle mixing. Data collection was performed at the University of Nebraska Medical Center Flow Cytometry Research Facility on a BD^TM^ LSRII flow cytometer (Franklin Lakes, NJ, USA) and analysis was done using FlowJo^TM^ v10.7 Software (Ashland, OR, USA).

### Global acetylation assay

The effects of M344 and vorinostat on acetylation in pancreatic cancer cells were determined with the EpiQuik^TM^ Global Histone H3 Acetylation Assay Kit (Epigentek, Farmingdale, NY, USA). Following the manufacturer’s instructions, histones were extracted from S2-013 cells that had been treated for 48 hours with 0.1% DMSO (vehicle control), or with 1 μM or 10 μM concentrations of M344 or vorinostat. The histones were spotted onto the wells, and then an antibody specific for acetylated histone H3 was added. After washing the wells, horseradish peroxidase-conjugated secondary antibody was added, followed by the detection reagent, and the resulting color change was measured with a BioTek 800 TS Absorbance Reader at 450 nm. A positive control (Acetylated Histone H3 Control) provided with the kit was included in the assay. Blank wells to which no histone proteins were added were also included. Percent acetylation was calculated as OD (treated sample–blank) / OD (untreated control–blank) X 100%.

### Immunoblotting

For immunoblotting, cells were lysed with buffer comprised of 50 mM Tris-HCl pH 7.5 (containing Sigma Trizma base), 1% Triton X-100 (Sigma), 1 mM EGTA (Sigma), 1 mM EDTA (Sigma), 2 mM DTT (Sigma), 1 mM Na_3_VO_4_ (Thermo Fisher Scientific), 0.1 mM PMSF (Sigma), and 1 μg/ml Halt Cocktail (Thermo Fisher Scientific). The lysates were frozen at -80°C overnight, then brought to 4°C on ice and centrifuged at 4°C for 30 minutes at 13,000 rpm. Samples of the cell lysate supernatants were combined with 5X sodium dodecyl sulfate loading dye, composed of 10% w/v sodium dodecyl sulfate (Tokyo Chemical Industry Company), 250 mM Tris-HCl pH 6.8, 5% v/v β-mercaptoethanol (Sigma), 30% v/v glycerol (Sigma), and 0.2% w/v bromophenol blue (Sigma). The mixtures were boiled for 5 minutes and electrophoresed on 4–20% Invitrogen Novex Tris-glycine polyacrylamide pre-cast gels (Thermo Fisher Scientific). After 2 hours of electrophoresis using 90 V at room temperature, the proteins were transferred to polyvinylidene difluoride Immobilon-P Millipore membranes at room temperature for 1 hour and 50 minutes using 30 V. After blocking for 1 hour at room temperature in 5% w/v nonfat dry milk, the membranes were incubated overnight with primary antibodies at 4°C. The membranes were then washed 3 times (for 5 minutes/wash) with 1% Tween-20 (Thermo Fisher Scientific) in PBS, incubated with secondary antibodies for 1 hour, and then washed 3 times with 1% Tween-20 in PBS (for 5 minutes/wash). The membranes were covered with Pierce ECL Western Blotting Substrate (Thermo Fisher Scientific), and the bands were visualized with the BioRad ChemiDoc Imaging System and Image Lab software (Bio-Rad Laboratories, Hercules, CA, USA).

### Flow cytometry

For flow cytometry, cells were removed from the plate with TrypLE^TM^ Express Enzyme (Thermo Fisher Scientific), resuspended in complete medium, and centrifuged at 1,500 rpm for 5 minutes at 4°C in an Eppendorf^TM^ 5810R centrifuge. Following centrifugation, the cells were resuspended in buffer (1X PBS containing 0.2% bovine serum albumin, 0.1% sodium azide) at a concentration of 5 x 10^6^/ml. Cells were transferred to a 96-well plate (at 100 μL/well) and centrifuged at 1,500 rpm for 5 minutes at 4°C in an Eppendorf^TM^ 5810R centrifuge. The cells were incubated with the primary antibody for 30 minutes on ice, washed with 1X PBS (3 times), incubated with fluorescently labeled secondary antibody for 30 minutes on ice, washed again in 1X PBS (3 times), fixed using 1% paraformaldehyde and analyzed on a BD^TM^ LSR II Flow Cytometer (BD Biosciences). Data collection was performed at the UNMC Flow Cytometry Research Facility and analysis was done using FlowJo^TM^ v10.7.

### Mouse tumor model and treatments

Female NU/J (athymic nude) mice (#002019), 4 weeks of age, were purchased from Jackson Laboratories (Bar Harbor, ME) and acclimated to their new environment for 2 weeks before they were used in experiments. The mice were housed in a pathogen-free facility at UNMC and analysis of M344 therapeutic effectiveness was performed according to Protocol #15-089-10-FC approved by the UNMC Institutional Animal Care and Use (IACUC) Committee. Orthotopic tumors were established by injecting S2-013 human pancreatic cancer cells into the pancreas of 6-week-old NU/J mice that had been anesthetized with isoflurane. Tumor volumes of anesthetized mice were monitored twice per week by ultrasound using the FUJIFILM VisualSonic Vevo^TM^ 3100 Imaging System (Toronto, ON, Canada), starting 8 days post-implantation. At 15 days post-implantation, the mice were randomized into treatment or control groups, with the groups having matched average tumor volumes. M344 was administered at 10 mg/kg via the intraperitoneal route for 5 days per week (5 days on treatment, 2 days off treatment). Gemcitabine was given every 3 days intraperitoneally at 50 mg/kg. At Day 25 post-tumor implantation, all mice were euthanized following the final tumor imaging while under anesthesia according to the American Veterinary Medical Association guidelines with cervical dislocation confirming animal death.

## Results

### M344 treatment interferes with pancreatic cancer cell proliferation

**Fig 1:**
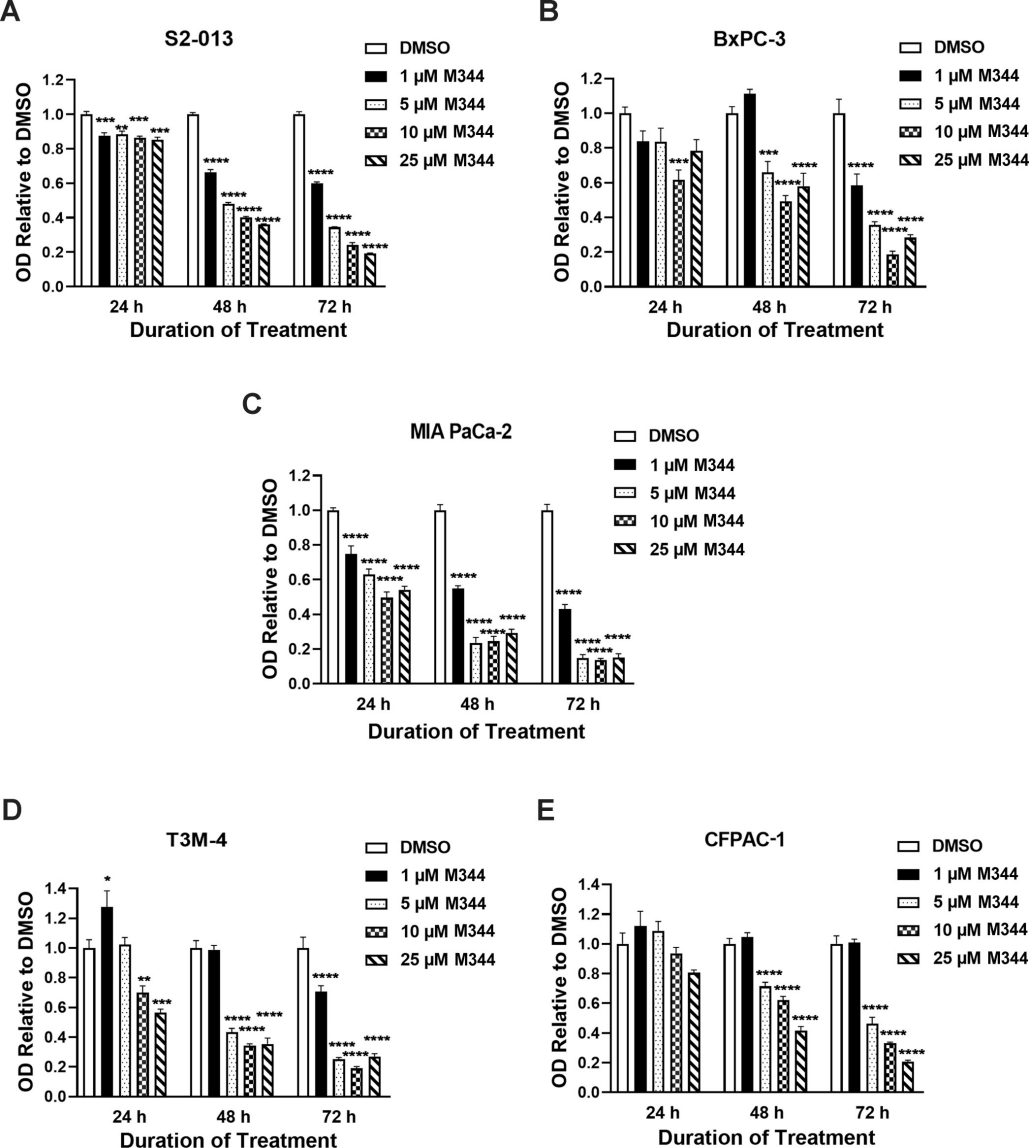
Pancreatic cancer cell proliferation is decreased upon M344 treatment. After treatment with M344 at various concentrations, the proliferation of (**A**) S2-013, (**B**) BxPC-3, (**C**) MIA PaCa-2, (**D**) T3M-4, and (**E**) CFPAC-1 cells was assessed by the MTT assay at 24, 48, and 72 hours. The graphs display the optical density (OD) at 570 nm relative to the OD for the 0.1% DMSO control. Each error bar represents the standard error of the mean. These results are representative of findings in 2–3 separate experiments for each cell line. The results from 0.1% DMSO control treatment versus treatment with each M344 concentration were compared using Ordinary One-way ANOVA with Dunnett’s Multiple Comparisons test in GraphPad Prism Version 8.4.2. The asterisks indicate the p values: * p<0.05, ** p<0.01, *** p<0.001, **** p<0.0001.

Pancreatic cancer cells are known for their proliferative, migratory, and metastatic capabilities, which all contribute to the pathogenesis of this disease [50, 51]. As an approach to monitor the effects of M344 on pancreatic cancer cells, the proliferation of M344-treated pancreatic cancer cells was investigated. Using the MTT assay, we found that at all M344 concentrations tested (1, 5, 10, and 25 μM) there was a statistically signficant reduction in the proliferation of S2-013 cells as compared to the 0.1% DMSO control (Fig 1A). The reduction in S2-013 proliferation became increasingly larger after prolonged periods (48 and 72 hours) (Fig 1A). BxPC-3 proliferation was also more diminished at 48 and 72 hours post-treatment than at 24 hours (Fig 1B). Among all the cell lines tested, MIA PaCa-2 was the most sensitive to M344 treatment, exhibiting significant decreases in proliferation at 24, 48, or 72 hours post-treatment with all concentrations of M344 evaluated (Fig 1C). As was observed with S2-013 and BxPC-3 cells, T3M-4 and CFPAC-1 proliferation decreased at 48 and 72 hours post-treatment (Fig 1D and 1E). CFPAC-1 proliferation was not significantly affected at 24 hours, but T3M-4 proliferation declined following treatment for 24 hours with 10 or 25 μM M344 (Fig 1D and 1E).

### M344 decreases pancreatic cancer cell viability

**Fig 2:**
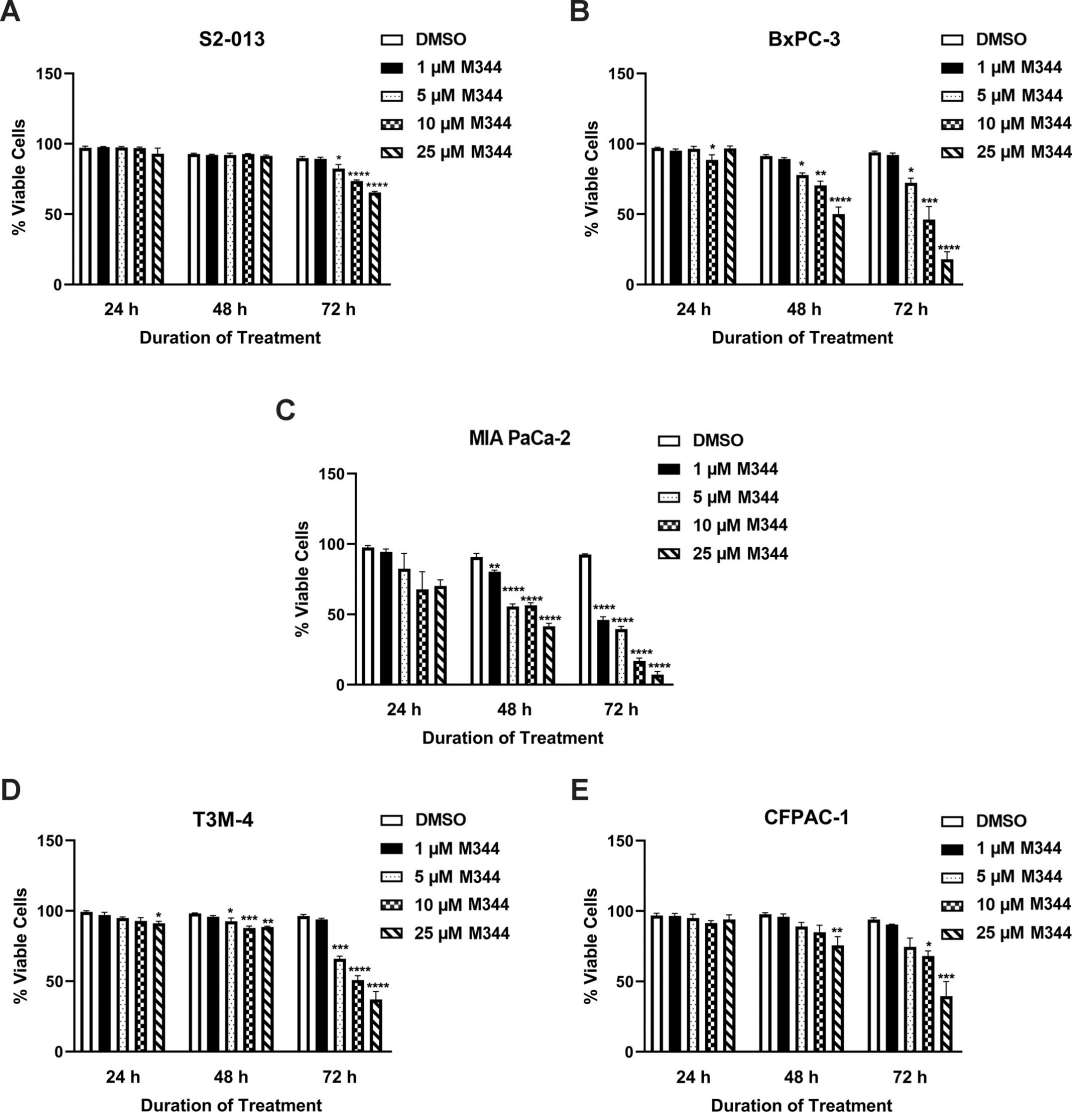
Pancreatic cancer cell viability is diminished by M344 treatment. After treatment with M344 at various concentrations, the viability of (**A**) S2-013, (**B**) BxPC-3, (**C**) MIA PaCa-2, (**D**) T3M-4, and (**E**) CFPAC-1 cells was assessed by the trypan blue exclusion assay at 24, 48, and 72 h. Viability was graphed as the number of live cells/total cells x 100. Each error bar represents the standard error of the mean. These results are representative of findings in 2–3 separate experiments for each cell line. The results from 0.1% DMSO control treatment versus treatment with each M344 concentration were compared using Ordinary One-way ANOVA with Dunnett’s Multiple Comparisons test in GraphPad Prism Version 8.4.2. The asterisks indicate the following p values: * p<0.05, ** p<0.01, *** p<0.001, **** p<0.0001.

Assessment by trypan blue staining of the effect of various M344 concentrations (1, 5, 10, and 25 μM) and treatment durations (24, 48, and 72 hours) on S2-013 cell viability showed a significant decrease at 72 hours (but not 24 or 48 hours) post-treatment with the higher M344 concentrations (Fig 2A). As with S2-013 cells, little to no effect on viability was observed for each of the other cell lines tested (BxPC-3, MIA PaCa-2, T3M-4, and CFPAC-1) after 24 hours of M344 treatment (Fig 2B–2E). At 48 and 72 hours, BxPC-3 cells had significantly decreased viability at most of the concentrations of M344 that were tested (Fig 2B), and MIA PaCa-2 cells showed a significant decrease at all of them (Fig 2C). The viability of T3M-4 and CFPAC-1 cells was reduced by the higher concentrations of M344 at both 48 and 72 hours (Fig 2D and 2E).

### M344 causes G_1_ arrest in pancreatic cancer cells

**Fig 3:**
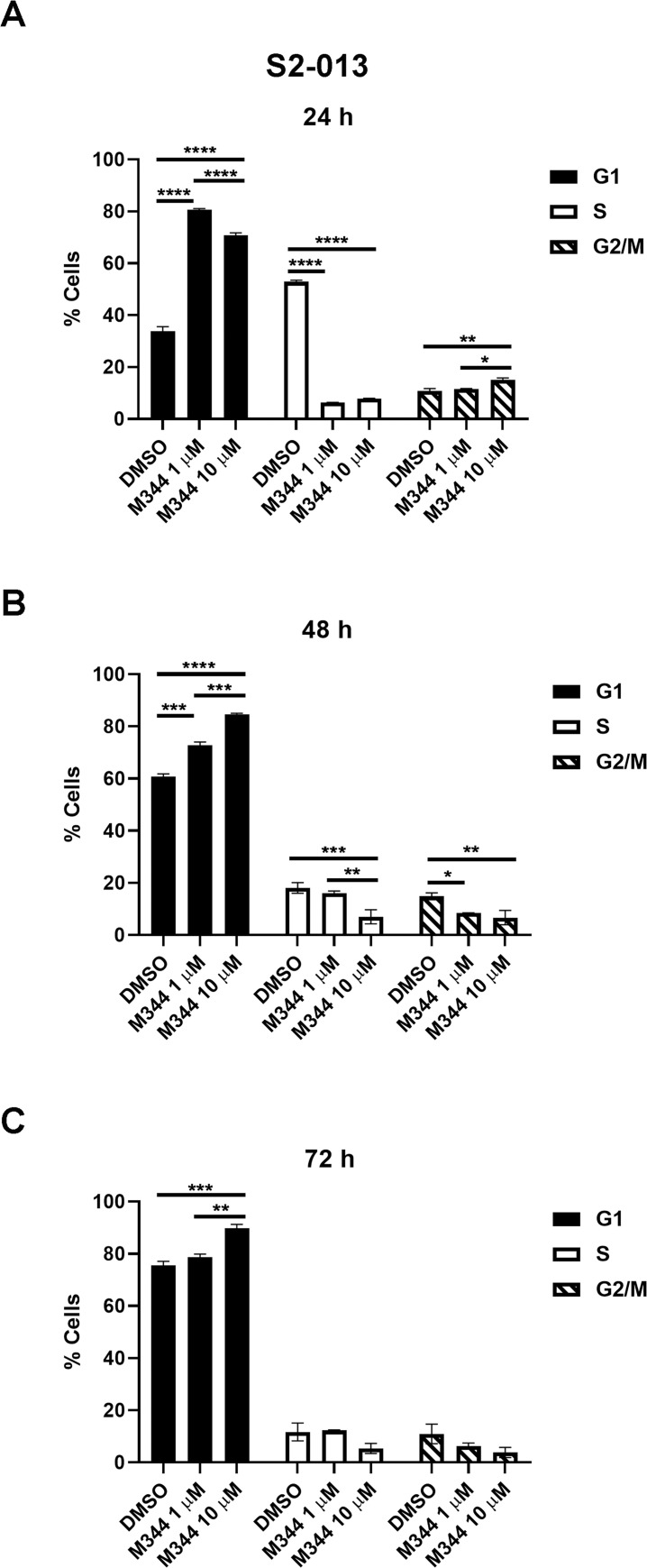
In pancreatic cancer cells, M344 causes cell cycle arrest in G_1_. Treatment of S2-013 cells with 1 or 10 μM M344 resulted in large increases in the populations accumulated in G_1_ at 24 hours (**A**), 48 hours (**B**), and 72 hours (**C**), as shown by propidium iodide staining and flow cytometry. Statistical comparisons were made using a Two-way ANOVA with Tukey’s Multiple Comparisons test in GraphPad Prism Version 8.4.2. The asterisks indicate the following p values: * p<0.05, ** p<0.01, ***p<0.001, **** p<0.0001.

To analyze M344’s mechanisms of action, we monitored the effects of M344 treatment on the pancreatic cancer cell cycle. S2-013 cells were treated for 24, 48, or 72 hours with M344 (1 or 10 *μ*M) or with 0.1% DMSO control. After permeabilization, the cells were stained with propidium iodide and DNA content was analyzed by flow cytometry. At 24 hours post-treatment with 1 or 10 *μ*M M344, the population of S2-013 cells in G_1_ was greatly increased in comparison to the 0.1% DMSO control (Fig 3A). Furthermore, at 24 hours the population of S2-013 cells in S phase dropped sharply after M344 treatment (at 1 or 10 μM), and there was a small but statistically significant increase in the G_2_/M population after 10 μM M344 treatment. By 48 hours (Fig 3B), the effects of M344 on the G1 and S populations were again similar but less accentuated. However, divergent from the 24-hour time point, a reduction in the G_2_/M population was observed at 48 hours for both 1 and 10 μM M344 compared to the 0.1% DMSO control. At the 72-hour time point, the extent of G_1_ arrest caused by 10 μM M344 was less in comparison to the 0.1% DMSO control (Fig 3C), likely due to the increased proportion of 0.1% DMSO control-treated cells in G_1_ as a result of increased confluency. It is notable that the rise in the G_1_ population induced by M344 (compared to the 0.1% DMSO control) occurs primarily within the first 24 hours of treatment and M344’s influence on cell cycle progression is an early event with lasting effects.

### By 48 hours, M344 at 10 μM causes apoptosis in pancreatic cancer cells

**Fig 4:**
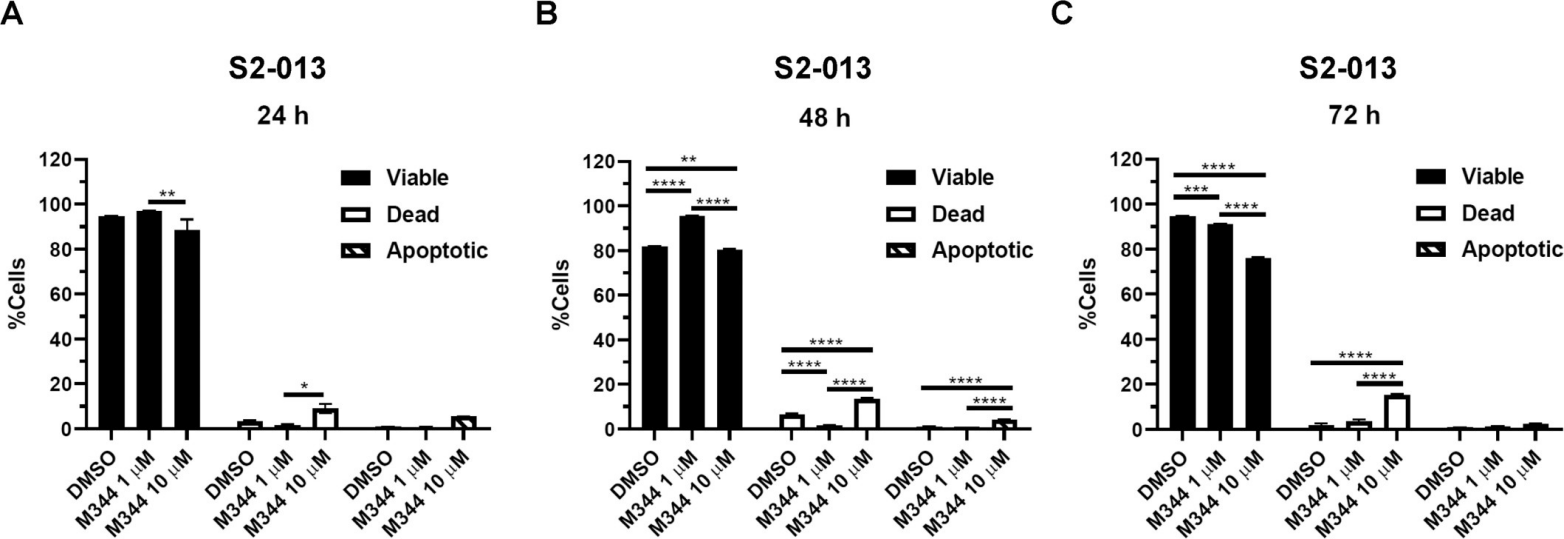
M344-induced apoptosis is apparent by 48 hours and necrosis peaks at 72 hours. S2-013 cells were treated with 0.1% DMSO control or M344 (1 μM or 10 μM) for 24, 48, or 72 hours. Caspase-3 and caspase-7 cleavage was simultaneously analyzed by using the CellEvent^TM^ Caspase-3/7 Green Flow Cytometry Assay Kit. The SYTOX^TM^ AADvanced^TM^ Dead Cell Stain included in the kit identified necrotic cells. Each error bar represents the standard error of the mean. Statistical comparisons of the results were done using a Two-way ANOVA with Tukey’s multiple comparisons test. The asterisks indicate the following p values: * <0.05, ** <0.01, *** <0.001, **** <0.0001.

To assess the mechanisms of action for M344 in greater detail, we analyzed the ability of M344 to induce apoptosis of pancreatic cancer cells. The cleavage of caspase-3 and -7 activates them, with the cleaved forms serving as apoptosis markers. After 24-, 48-, or 72-hour treatment of S2-013 cells with M344 (1 or 10 μM) or 0.1% DMSO control, we used the CellEvent^TM^ Caspase-3/7 Green Flow Cytometry Assay Kit to analyze the cells for the presence of cleaved caspase-3 and -7. Fig 4 shows graphs of the data from triplicate samples with statistical analysis, and an example of the gating strategy is shown in S2 Fig. The apoptotic and dead cell populations were not significantly increased at 24 hours post-treatment with M344 in comparison to the 0.1% DMSO control (though there was a slight, significant rise in the percentage of dead cells after treatment with 10 μM M344 compared with 1 μM M344 at that time point) (Fig 4A). However, at 48 hours, apoptotic and dead cells were increased by the 10 μM M344 treatment (p<0.0001) (Fig 4B). By 72 hours after treatment with either 1 or 10 μM M344, the percentage of dead cells was increased compared to the 0.1% DMSO control (p<0.0001) (Fig 4C) and was higher than at 24 or 48 hours (Fig 4A and 4B).

### M344 treatment slows pancreatic cancer cell migration

**Fig 5:**
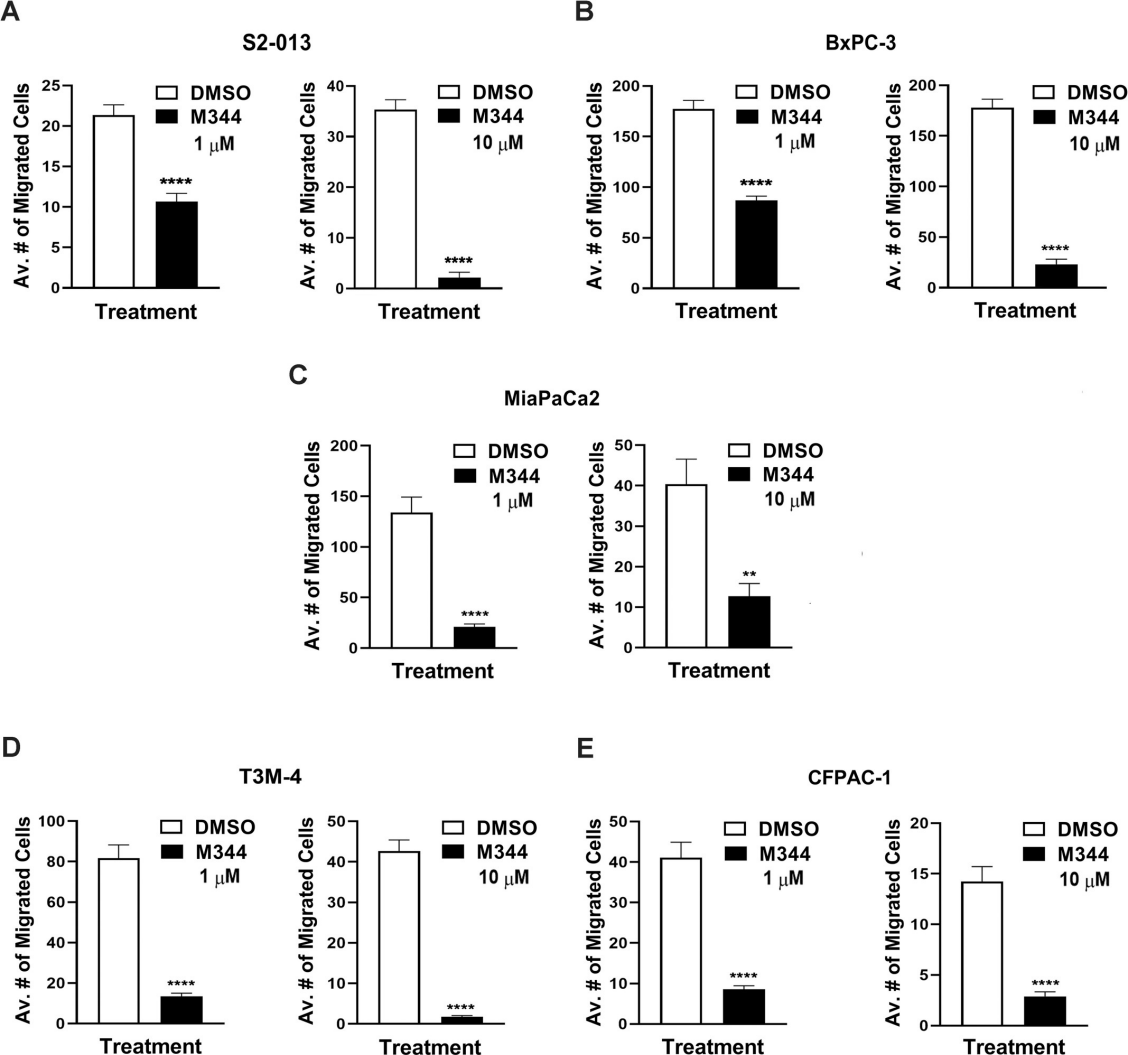
Migration of pancreatic cancer cells is reduced upon treatment with M344. Transwell assays were performed to assess the migration of (**A**) S2-013, (**B**) BxPC-3, (**C**) MIA PaCa-2, (**D**) T3M-4, and (**E**) CFPAC-1 cells following treatment with 1 μM or 10 μM of M344 (or with 0.1% DMSO, as the vehicle control) for 24 h. For the transwell assays, after treatment the cells were plated into 3 separate 8-μm inserts (1x10^5^ cells/insert) in the presence of 1 μM M344, 10 μM M344, or 0.1% DMSO and incubated for 24 h. Cells were fixed and stained, and photographs of 3 random fields were taken for each of the inserts. The results of counts of the stained, migrated cells were averaged. The graph shows the average numbers of cells that migrated, and each error bar represents the standard error of the mean. The presented graph for the S2-013 cells is representative of the results from 3 separate experiments; the full experimental set of counts for each of the other cell lines was done once. The results of 0.1% DMSO control treatment versus the 1 μM or the 10 μM M344 treatment were compared using Student’s *t* test. The asterisks indicate the following p values: * p<0.05, ** p< 0.01, *** p<0.001, and **** p<0.0001.

The ability of pancreatic cancer cells to actively migrate can contribute to their metastasis [51]. Treatment of pancreatic cancer cell lines with either 1 μM or 10 μM M344 greatly reduced the ability of S2-013, BxPC-3, MIA PaCa-2, T3M-4, and CFPAC-1 cells to migrate in a transwell assay (Fig 5A–5E; migration data images are shown in the S1 Fig). Furthermore, the migration of the S2-013 and T3M-4 cells was almost completely abrogated by the 10 μM M344 treatment (Fig 5A and 5D). It should be noted that M344 at 1 μM does not have a statistically significant effect on the viability of any of these cell lines other than MIA PaCa-2 (as was shown earlier in Fig 2C), indicating that the decreased cell migration detected following a 1 μM M344 treatment is not merely due to cell death. These data clearly indicate that M344 has the effect of lessening pancreatic cancer cell migration, which could diminish metastasis.

### M344 reduces the proliferation of pancreatic cancer cells better than vorinostat

**Fig 6:**
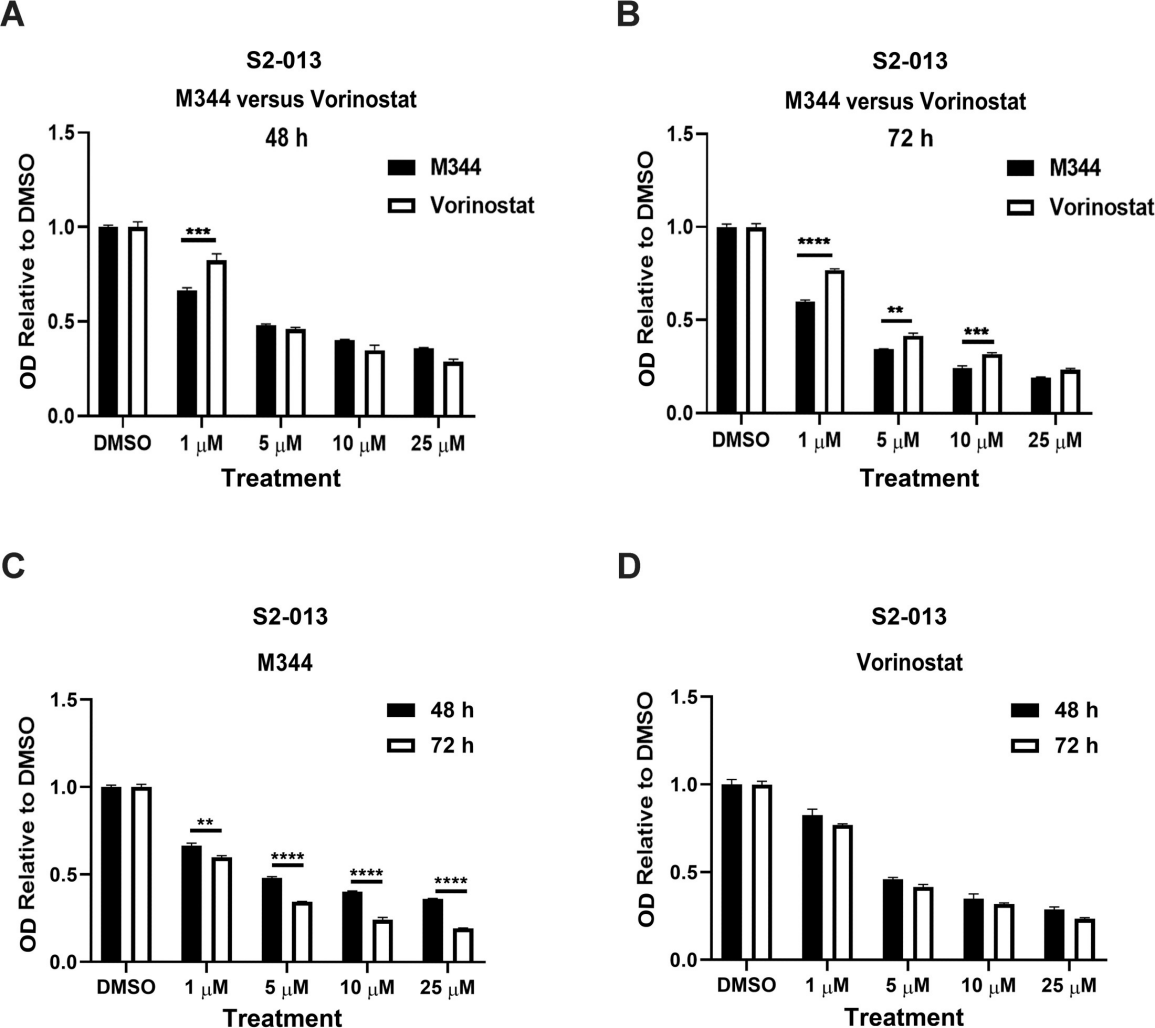
Compared to vorinostat, M344 decreases pancreatic cancer cell proliferation more effectively for a longer duration. The proliferation of S2-013 cells was assessed by the MTT assay following treatment with 0.1% DMSO control or with 1 μM, 5 μM, 10 μM, or 25 μM M344 or vorinostat for 48 hours or 72 hours. The effects of M344 versus vorinostat at 48 hours (**A**) and at 72 hours (**B**) are shown. The same data are displayed for M344 (**C**) and vorinostat (**D**) at 48 hours versus 72 hours. The error bars represent the standard error of the mean. The results were compared using a Two-way ANOVA with Tukey’s multiple comparisons test in GraphPad Prism Version 8.4.2. The asterisks indicate the following p values: ** p< 0.01, *** p<0.001, **** p<0.0001.

To determine the effectiveness of M344 relative to the clinically utilized HDAC inhibitor vorinostat, we compared the impact of these two inhibitors on S2-013 cell proliferation over a range of treatment concentrations and durations. At 48 hours, M344 had a better anti-proliferative effect than vorinostat at a low concentration (1 μM) (Fig 6A), and at 72 hours significant differences were noted at several concentration levels (Fig 6B). At 72 hours after treatment with 1 μM of M344 or vorinostat, we observed the greatest difference between M344 and vorinostat in anti-proliferative activity, as S2-013 proliferation was reduced by 40% with M344 treatment but only by 23% with vorinostat (Fig 6B). Likewise, at 5 μM and 10 μM, the proliferation of S2-013 cells decreased more after treatment with M344 than vorinostat (at 5 μM by 66% versus 58%, and at 10 μM by 76% versus 68%). We also graphed the same data shown Fig 6A and 6B in a different format in Fig 6C and 6D to show the capacity of M344 or vorinostat to affect pancreatic cancer cell proliferation over time. M344 treatment led to a significant reduction in S2-013 cell proliferation between the 48-hour and 72-hour treatment time points at all M344 concentrations tested (Fig 6C). In contrast, for S2-013 cells treated with vorinostat, the abatement in proliferation was not significantly different between the 48- and 72-hour time points at any of the vorinostat concentrations evaluated (Fig 6D). These results suggest that M344 is not only a more potent inhibitor of pancreatic cancer cell proliferation than vorinostat, but that it also has more prolonged anti-proliferative effects on these cells.

### M344 is more effective than vorinostat at inhibiting histone deacetylation in pancreatic cancer cells

**Fig 7:**
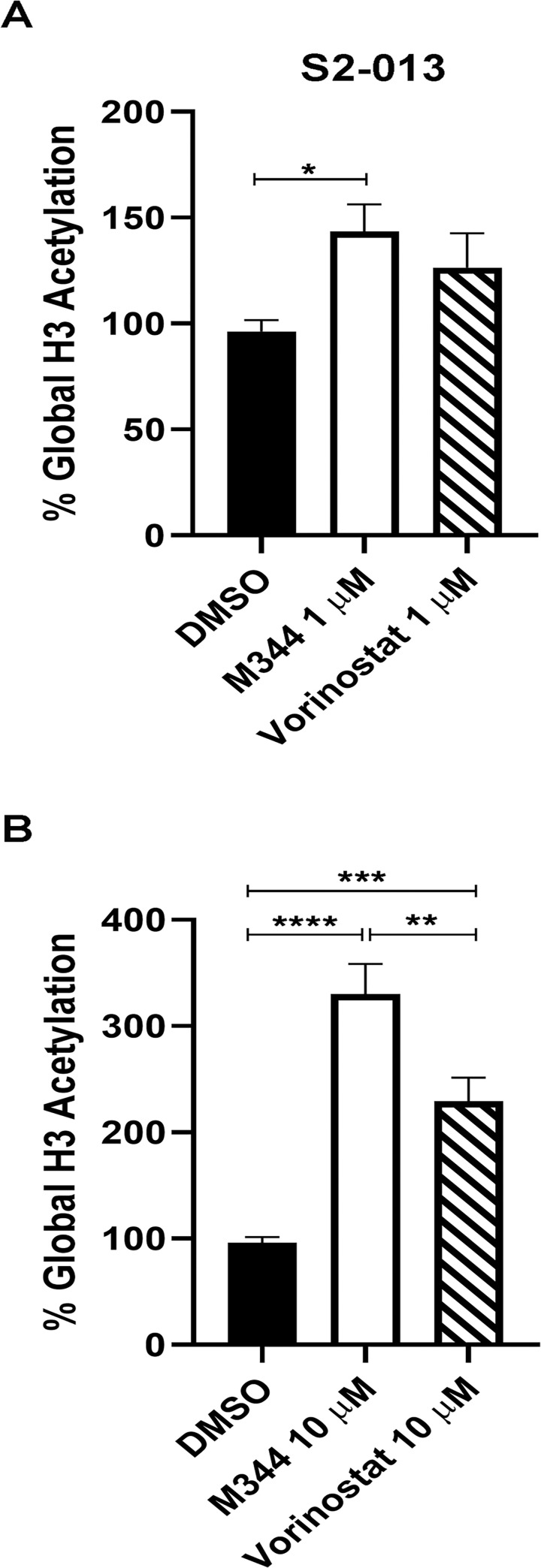
M344 treatment of pancreatic cancer cells increases global histone H3 acetylation more than vorinostat treatment. The inhibition of histone H3 deacetylation in S2-013 cells by M344 versus vorinostat was compared by monitoring the percentage of acetylated histone H3 following 48-hour treatment with M344 or vorinostat (at 1 μM or 10 μM) or with the 0.1% DMSO vehicle alone. Global H3 acetylation was assessed using the EpiQuik^TM^ Global Histone H3 Acetylation Assay Kit. The data are displayed in (**A**) for 1 μM M344 and vorinostat (and 0.1% DMSO control), and in (**B**) for 10 μM M344 and vorinostat (and 0.1% DMSO control). These data were compiled from two biological replicates with triplicate samples in the first assay and quintuplet samples in the second assay. The graphs display the percentage global histone H3 acetylation, calculated by this formula: OD (treated sample–blank) / OD (untreated control–blank) X 100%. Error bars represent the standard error of the mean. Statistical comparisons were made with One-Way ANOVA with Tukey’s multiple comparisons tests. The asterisks indicate the p values: * p<0.05, ** p<0.01, *** p<0.001, **** p<0.0001.

Since our findings indicated that M344 has more potent and durable effects on pancreatic cancer cell proliferation than vorinostat, we next compared the capability of M344 and vorinostat to inhibit deacetylation. S2-013 cells were treated for 48 hours with M344 or vorinostat at 1 μM or 10 μM or were treated only with the vehicle (0.1% DMSO) as a control, and acetylation was assayed using an EpiQuik Global Histone H3 Acetylation Assay Kit. At 1 μM, M344 (but not vorinostat) increased S2-013 H3 acetylation (Fig 7A). At 10 μM, either drug was able to increase global H3 acetylation, but 10 μM M344 caused a greater increase than 10 μM vorinostat (Fig 7B).

### M344 and vorinostat have similar effects on the viability of non-malignant cells

**Fig 8:**
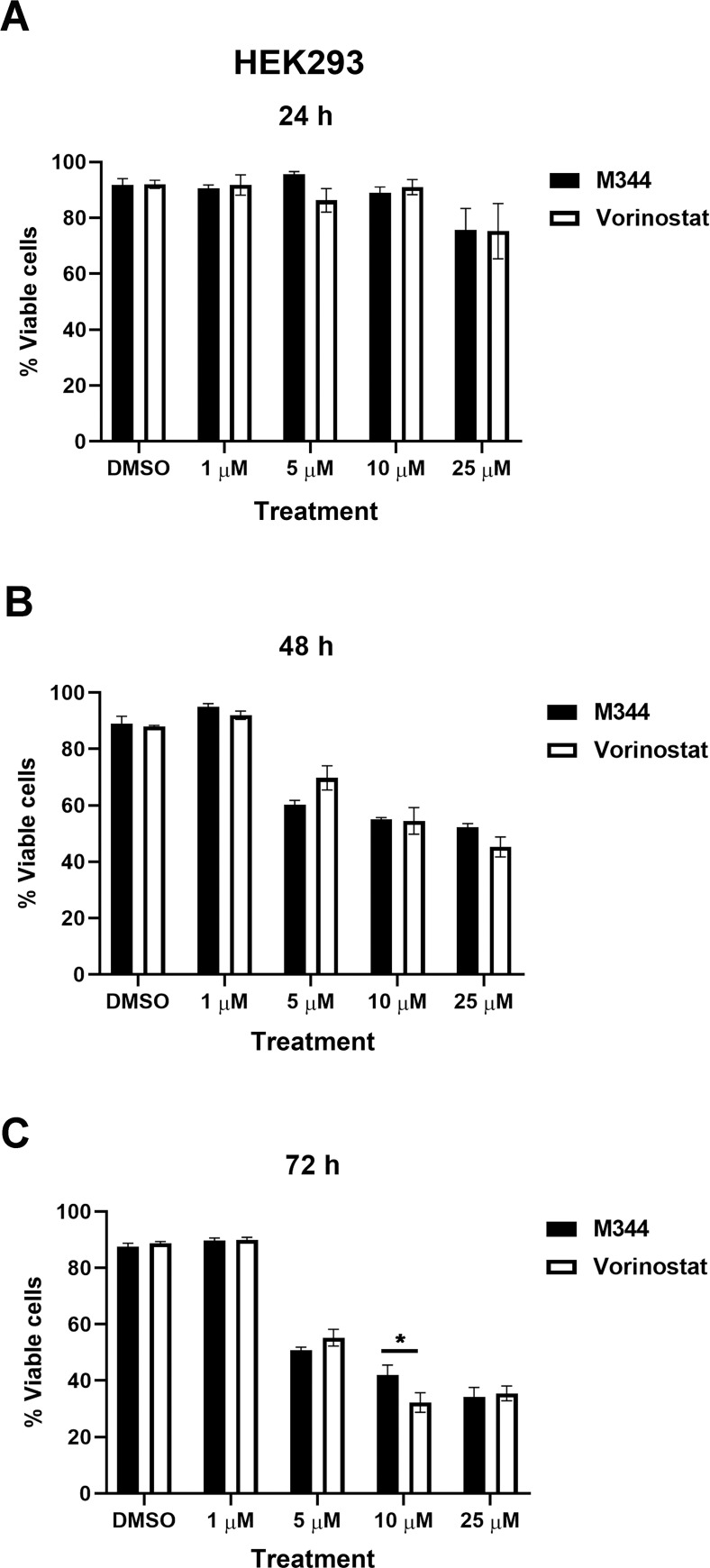
M344 does not impair HEK293 cell viability more than vorinostat. After treatment with M344 or vorinostat at the indicated concentrations, the viability of HEK293 cells was assessed by the trypan blue exclusion assay at 24, 48, or 72 hours. Viability was graphed as the number of live cells/total cells x 100. Each error bar represents the standard error of the mean. The results from the 0.1% DMSO control treatment versus treatment with each M344 and vorinostat concentration were compared using a Two-way ANOVA with Sidak’s multiple comparisons test. The asterisks indicate the p value: * p<0.05.

To assess whether M344 is comparable to vorinostat regarding toxicity in non-cancerous cells, we tested both inhibitors against HEK293 human embryonic kidney cells. As shown in Fig 8, there were no significant differences between the effects of vorinostat and M344 on HEK293 cells, with the exception that 10 *μ*M M344 for 72 hours impaired viability less than the same concentration of vorinostat. Thus, these results suggest that M344 does not diminish non-malignant cell viability more than vorinostat, which (as noted above) is already FDA approved for cutaneous T cell lymphoma treatment [11].

### M344 treatment of pancreatic cancer cells augments the expression of the MHC class I heavy chain protein and the surface expression of the MHC class I molecule

**Fig 9:**
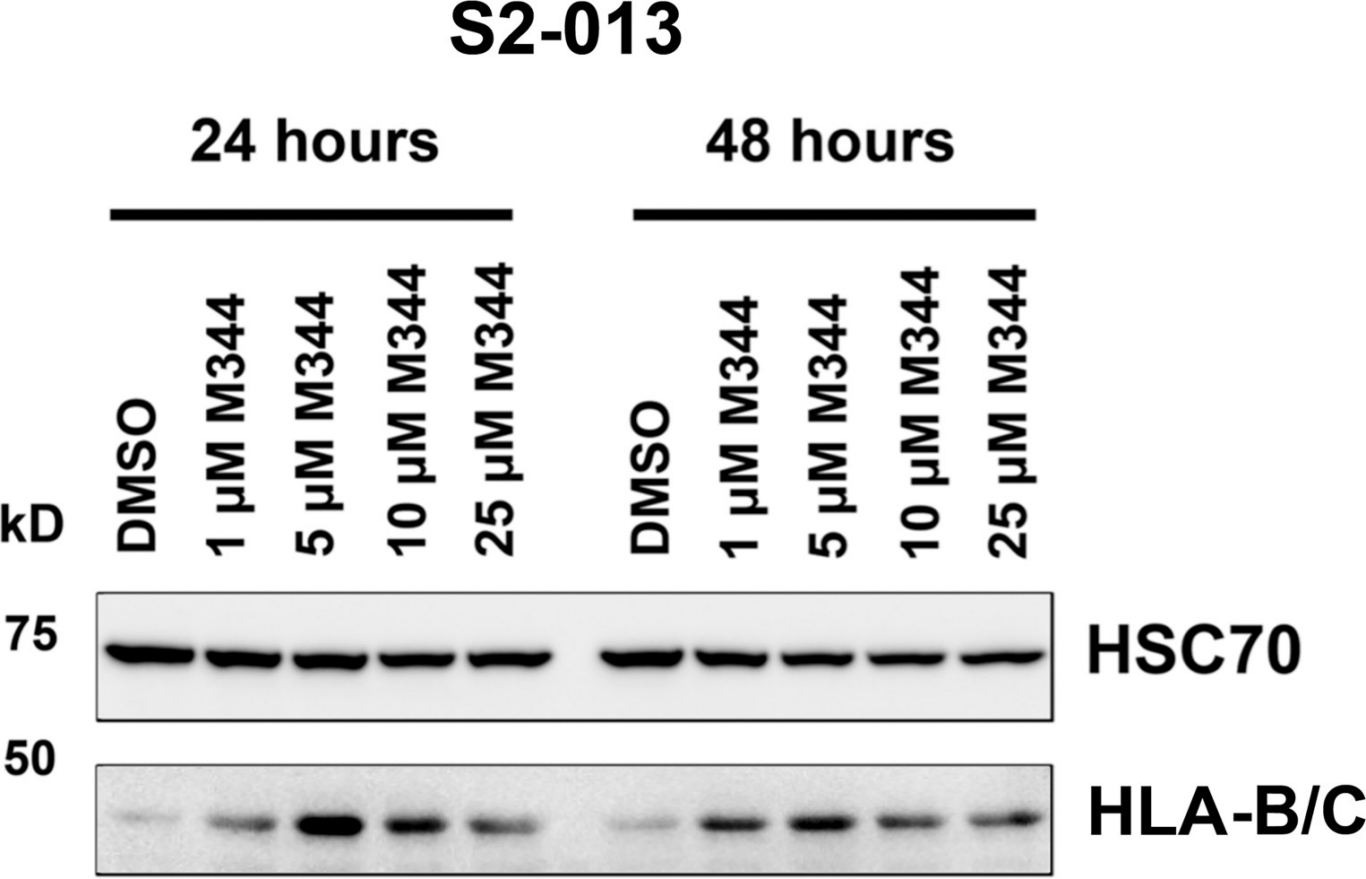
M344 treatment increases the expression of the human MHC class I heavy chains HLA-B and -C. For immunoblot analysis, lysates of S2-013 pancreatic cancer cells treated for 24 or 48 hours with 0.1% DMSO (vehicle control) or with 1, 5, 10, or 25 μM M344 were subjected to electrophoresis, and the proteins were transferred to a membrane that was cut and one section was probed with an antibody for HSC70 (as a loading control) and another section was probed with an antibody to detect the MHC class I heavy chain (specifically, with the HC10 antibody that binds to HLA-B and HLA-C heavy chains). The exposure for the HLA-B/C heavy chains that is shown in the figure is longer than the exposure for the HSC70 control. Similar results were also obtained using the same M344 concentrations, time points, and antibodies in a separate experiment using different S2-013 lysates.

Previous investigations of the effects of HDAC inhibitors on cancer cells have included exploration of the impact of these inhibitors on the expression of MHC class I molecules, and in several instances positive effects have been noted [e.g., 18, 19, 26, 27]. Fully assembled MHC class I molecules are heterotrimers of MHC class I heavy chain, beta 2-microglobulin, and peptide antigen. The function of MHC class I molecules is to present peptide antigens (such as fragments of tumor-associated proteins) to cytotoxic T lymphocytes. Antigen presentation by MHC class I molecules can trigger T cells to kill the target cells expressing the antigens by release of granzyme and perforin. However, when there is reduced expression of MHC class I molecules by tumor cells, the vulnerability of those cells to lysis by cytotoxic T cells is lessened [52–54]. To determine if the expression of MHC class I heavy chain proteins in pancreatic cancer cells is increased by M344 treatment, we performed immunoblot analysis on lysates of M344-treated S2-013 pancreatic cancer cells with an antibody recognizing the human MHC class I heavy chains HLA-B and HLA-C. HSC70 protein was also monitored as a loading control. As shown in Fig 9 and S3 Fig, the expression of the MHC class I heavy chain was upregulated in S2-013 cells at 24 hours and at 48 hours post-treatment with M344. Thus, M344 treatment increases the expression of the MHC class I heavy chain total protein in S2-013 pancreatic cancer cells.

**Fig 10:**
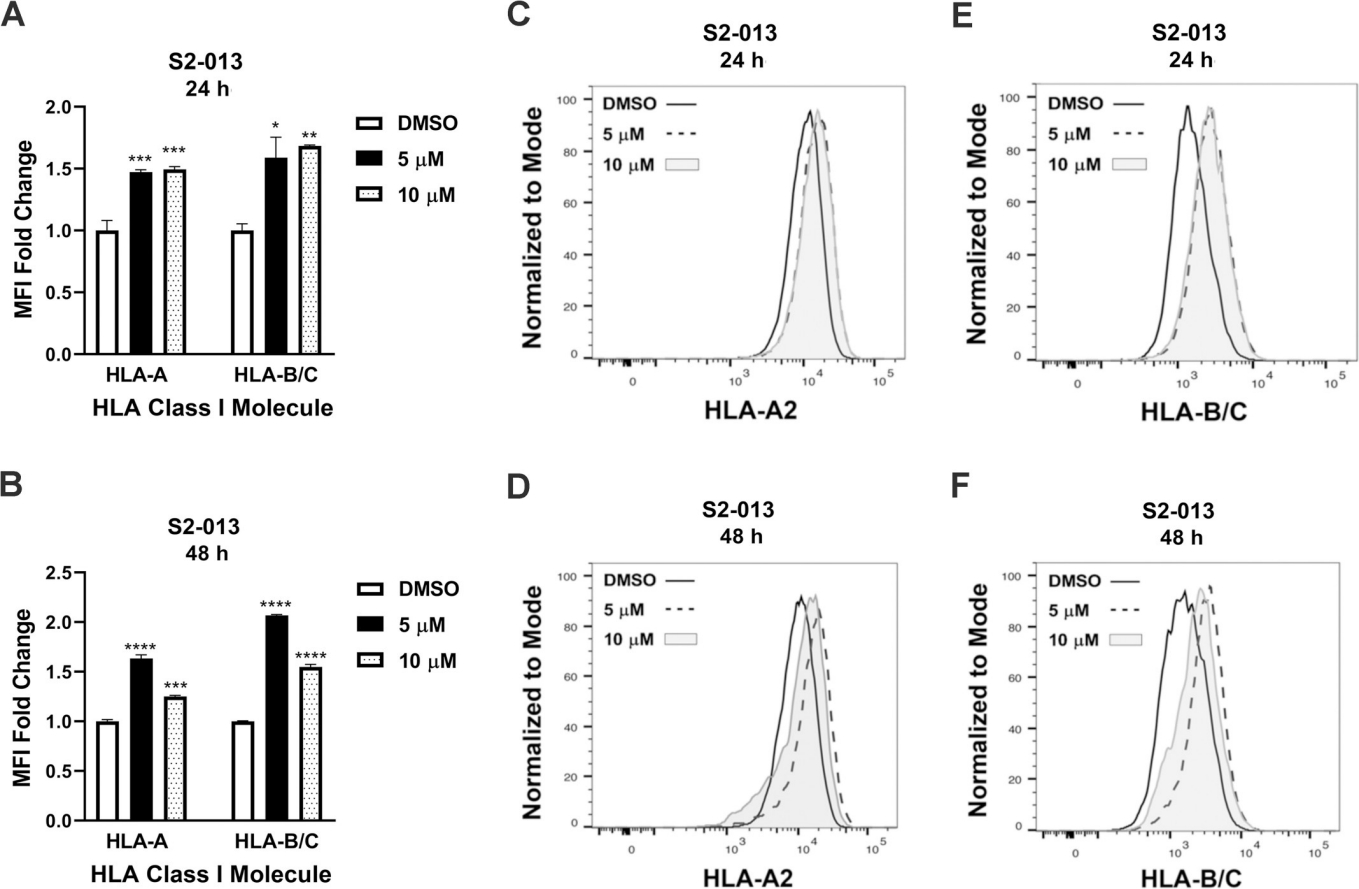
MHC class I expression on S2-013 pancreatic cancer cells is increased following M344 treatment. After 24- or 48-hour treatments with 0.1% DMSO (vehicle control) or with M344 at 5 or 10 μM concentrations, cell surface expression was monitored using flow cytometry with the BB7.2 antibody for peptide-occupied HLA-A2 and the B1.23.2 antibody that detects HLA-B/C. The bar graphs depict the median fluorescence intensity (MFI) fold change relative to the 0.1% DMSO control treatment for (**A**) 24-hour M344 treatment and (**B**) 48-hour M344 treatment. Triplicate wells for each concentration and time point were analyzed. Each error bar represents the standard error of the mean. Statistical comparison of the results from the 0.1% DMSO control treatment versus treatment with each M344 concentration was performed using Ordinary One-way ANOVA with Dunnett’s Multiple Comparisons Test in GraphPad Prism Version 8.4.2. The asterisks indicate the p values: * p<0.05, ** p< 0.01, *** p<0.001, **** p<0.0001. Representative histogram overlays are shown for S2-013 (**C**) HLA-A2 expression at 24 hours post-treatment with M344, (**D**) HLA-A2 at 48 hours post-treatment with M344, (**E**) HLA-B/C at 24 hours post-treatment with M344, and (**F**) HLA-B/C at 48 hours post-treatment with M344. In the histograms, solid lines represent 0.1% DMSO treatment, dashed lines represent 5 μM M344 treatment, and solid gray areas represent 10 μM M344 treatment.

Because the effective function of MHC class I molecules depends on their presence at the plasma membrane in position to present peptide antigens to T cells, we also undertook studies of the influence of M344 treatment on the level of MHC class I molecules at the cell surface. Using flow cytometry, we monitored MHC class I expression with the BB7.2 antibody [45, 46] that recognizes peptide-bound HLA-A2 (an MHC class I molecule that is expressed by S2-013 pancreatic cancer cells [55]), and with an antibody that recognizes HLA-B and HLA-C (B1.23.2) [47]. Treatment of S2-013 cells with M344 at either a 5 μM or 10 μM concentration for 24 hours or 48 hours enhanced the expression of MHC class I molecules at the plasma membrane (Fig 10). These findings indicate that M344 treatment results in heightened preparedness of the S2-013 cells to present tumor antigens to cytotoxic T lymphocytes.

### M344 and gemcitabine in combination decrease pancreatic cancer cell proliferation effectively and M344 complements gemcitabine’s ability to reduce pancreatic cancer cell viability

**Fig 11:**
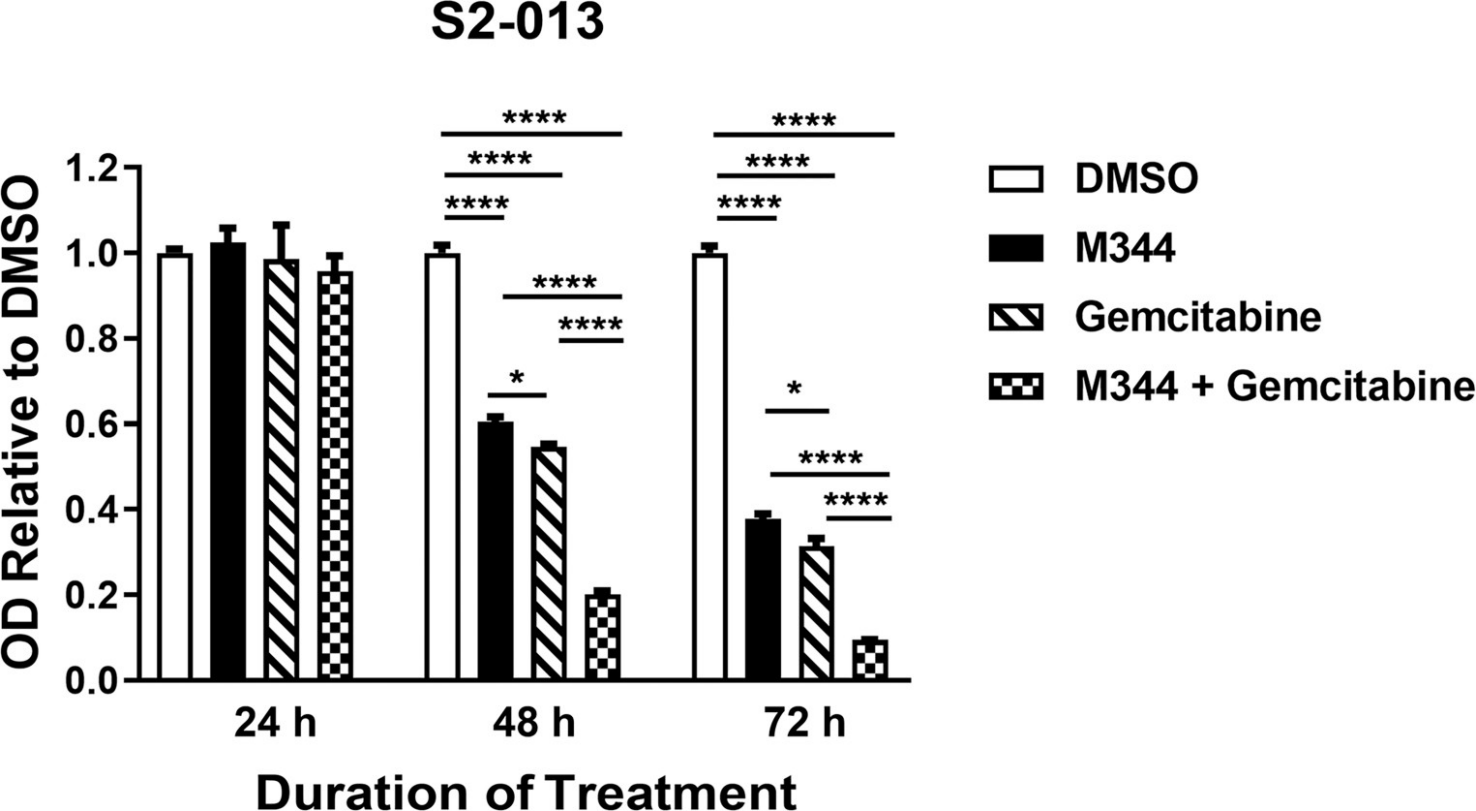
S2-013 pancreatic cancer cell proliferation is decreased by treatment with the combination of M344 and gemcitabine more than by either treatment alone at 48 or 72 hours post-treatment. The proliferation of S2-013 cells following treatment with DMSO, 10 μM M344, 100 nM gemcitabine, or 10 μM M344 + 100 nM gemcitabine was monitored by MTT assay at the indicated time points. These results are representative of the data from 4 separate experiments. The error bars represent the standard error of the mean. The results were compared using Ordinary One-way ANOVA with Tukey’s Multiple Comparisons test in GraphPad Prism Version 8.4.2. The asterisks indicate the p values: * p<0.05, ** p< 0.01, *** p<0.001, **** p<0.0001.

**Fig 12:**
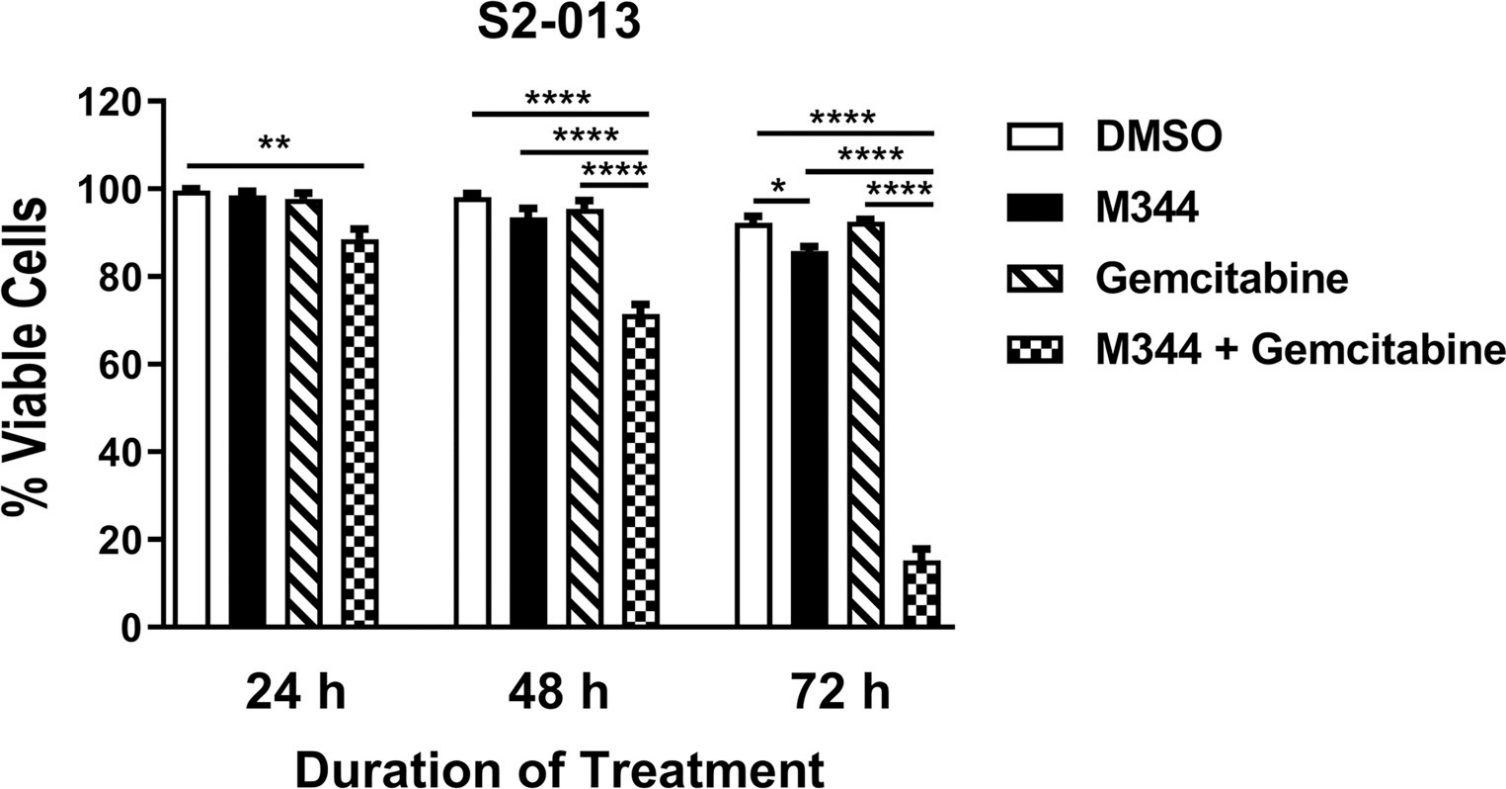
M344 impairs viability in combination with gemcitabine. S2-013 pancreatic cancer cells were treated with 0.1% DMSO, 10 μM M344, 100 nM gemcitabine, or 10 μM M344 + 100 nM gemcitabine. Viability was assessed by the trypan blue exclusion assay at 24, 48, and 72 hours and graphed as the number of live cells/total cells x 100. Each error bar represents the standard error of the mean. The results from 0.1% DMSO control treatment versus treatment with each M344 concentration were compared using Ordinary One-way ANOVA with Dunnett’s Multiple Comparisons test in GraphPad Prism Version 8.4.2. The asterisks indicate the p values: * p<0.05, ** p< 0.01, **** p<0.0001.

We sought to observe the effects on pancreatic cancer cell growth following combined M344 and gemcitabine treatment, since gemcitabine is commonly used as a first-line treatment for patients with pancreatic cancer. By the MTT assay, we demonstrated a significant reduction in the proliferation of S2-013 cells cultured with both 10 μM M344 and 100 nM gemcitabine, as compared to 0.1% DMSO control or either of the individual drugs, at 48 and 72 hours post-treatment (Fig 11). Furthermore, using trypan blue staining, we showed that S2-013 cells treated with both drugs in combination had lower viability than S2-013 cells treated with 0.1% DMSO, M344, or gemcitabine alone at 48 and 72 hours post-treatment (Fig 12). The decrease in S2-013 viability was particularly evident at 72 hours after the combination treatment. Specifically, the S2-013 cells had only 15% viability after M344 + gemcitabine treatment compared to 92% viability after DMSO treatment (Fig 12).

### M344 treatment slows pancreatic tumor growth in a mouse model

**Fig 13:**
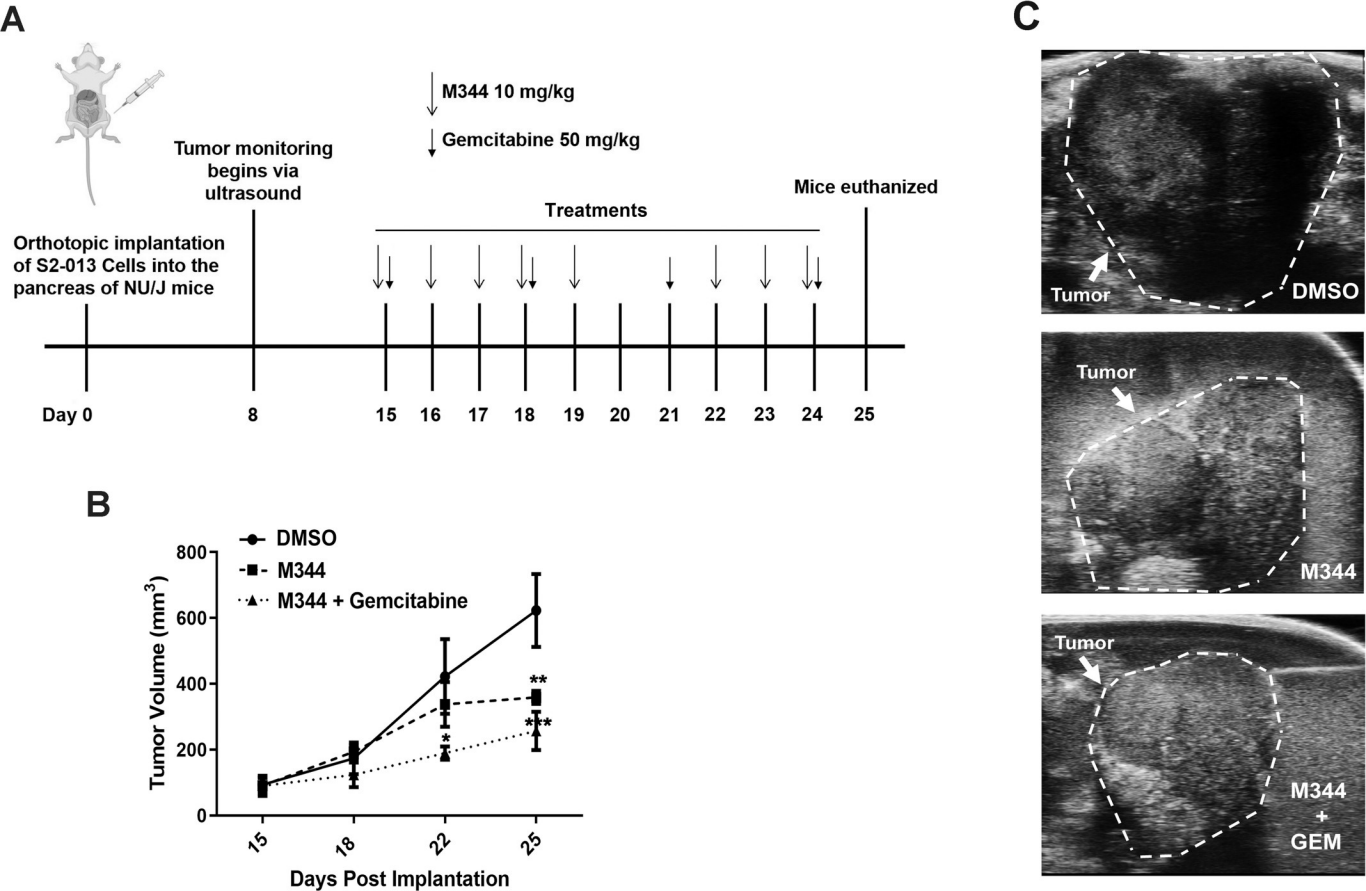
M344 decreases orthotopic pancreatic tumor growth when used as a treatment alone or in combination with gemcitabine. (**A**) S2-013 cells were orthotopically implanted into the pancreas of female NU/J mice. After 8 days, the tumor volume for each mouse was monitored twice weekly with the VisualSonic Vevo 3100 Imaging System. At 15 days post-implantation of tumor cells, the mice were randomized into control or treatment groups with matched average tumor volumes. M344 was administered intraperitoneally at 10 mg/kg for 5 days per week (5 days on, 2 days off). Gemcitabine was given every 3 days intraperitoneally at 50 mg/kg. On Day 25 post-tumor implantation, the mice were euthanized and the tumors were resected and weighed. The changes in tumor volume over time are shown in (**B**) and representative images of tumors at 25 days post implantation are shown in (**C**). For statistical analysis, ordinary One-way ANOVA with Dunnett’s Multiple Comparisons test in GraphPad Prism Version 8.4.2 was used. The asterisks indicate the following p values: * p<0.05, ** p< 0.01, *** p<0.001.

To assess the effect of M344 on pancreatic tumors in a xenograft mouse model, S2-013 pancreatic cancer cells were injected into the pancreas of nude mice. At Day 15 following orthtopic S2-013 cell implantation, the mice were separated into groups with matched average tumor volumes (based on ultrasound imaging). The groups were treated with vehicle control (5% Tween and 2.5% DMSO in saline), M344 (10 mg/kg), or M344 (10 mg/kg) + gemcitabine (50 mg/kg) (Fig 13A). By Day 22, the tumors of mice treated with M344 + gemcitabine had significantly smaller volumes than the tumors of mice treated with vehicle control only (Fig 13B). Furthermore, by Day 25, the volumes of the tumors in mice treated with either M344 alone or with M344 + gemcitabine were significantly reduced compared to the tumor volumes of vehicle control-treated mice. This outcome, coupled with our results obtained in the pancreatic cancer cell model studies (as described above), support further investigation of M344 as a possible pancreatic cancer therapy in studies with larger mouse cohorts and a comparison of M344 alone versus gemcitabine alone as individual pancreatic tumor treatments.

## Discussion

Several HDAC inhibitors, including vorinostat, have shown encouraging results as potential new therapies in pancreatic cancer studies [2, 5–10, 12, 13, 56–60]. M344 inhibits HDACs 1, 2, 3, 6, and 10 to a similar extent as vorinostat, and M344 also inhibits some additional HDACs more effectively than vorinostat [11, 29]. In good accordance with our current discoveries regarding M344, data by Laschanzky et al. demonstrated that inhibition of HDACs 1, 2, and 6 by other HDAC inhibitors effectively complements the therapeutic effects of gemcitabine against pancreatic cancer [14].

Since vorinostat has an established safety record and is FDA approved [11], our finding that M344 does not decrease HEK293 cell viability more than vorinostat is an important step toward consideration of its future use as a pancreatic cancer treatment. M344 can be administered to mice for at least 4 months without evidence of adverse events [29]. The relatively low *in vivo* toxicity of M344 is a notable factor for this HDAC inhibitor that could facilitate its entry into clinical trials.

Our findings indicate that M344 inhibits the proliferation, survival, and migration of pancreatic cancer cells. M344’s effects on proliferation in our experiments are consistent with a report by Furchert et al. showing M344 could inhibit the growth of pediatric embryonal tumors of the nervous system [30]. M344 was previously noted to enhance cisplatin killing of breast and ovarian cancer cell lines [61], and our data suggest that it also complements gemcitabine as a chemotherapeutic for pancreatic cancer. In addition, M344’s ability to upregulate total and cell-surface MHC class I expression on pancreatic cancer cells suggests that it may have beneficial effects if administered to patients concurrently with immunotherapy. MHC class I expression on tumor cells is recognized as important for the success of immune checkpoint inhibitor therapy for cancer patients [62]. This known contribution of MHC class I molecule expression to immune checkpoint inhibitor therapy efficacy is consistent with recent reports that document the ability of HDAC inhibitors to facilitate immune checkpoint inhibitor effectiveness when the two are administered in the same treatment regimen [26, 63]. Since gemcitabine also has favorable immunomodulatory effects [64–66], M344 + gemcitabine should be evaluated for cumulative impact on anti-pancreatic tumor immunity. Taken together, our results support further development of the HDAC inhibitor M344 as a novel treatment for pancreatic cancer, as well as investigation into its potential therapeutic impact when used against other cancers.

## Supporting information

S1 Fig Migration of pancreatic cancer cells after M344 treatment. These transwell migration assay data correspond to the graph that is displayed in Fig 5, and the experimental details are listed in the legend for Fig 5.
(TIF)

S2 Fig Example of flow cytometry gating for the caspase 3/7 activation assay. Details of the experimental process can be found in the legend for Fig 4.
(TIF)

S3 Fig Immunoblotting of pancreatic cancer cell line HLA class I heavy chains following treatment of the cells with M344. The immunoblot data displayed here correspond to Fig 9. The proteins were transferred after electrophoresis to a membrane that was then divided. The top portion was probed with anti-HSC 70 (loading control) antibody and the bottom portion was probed with the HC10 antibody (for HLA-B and–C heavy chains). The blots were imaged, and a long exposure and a short exposure are shown (on the left and right, respectively).
(TIF)
