# Supplementary figures and images for: The histone deacetylase inhibitor M344 as a multifaceted therapy for pancreatic cancer
Source: PLoS One. 2022 Sep 20;17(9):e0273518. doi: 10.1371/journal.pone.0273518 (PMC9488834; doi:10.1371/journal.pone.0273518)

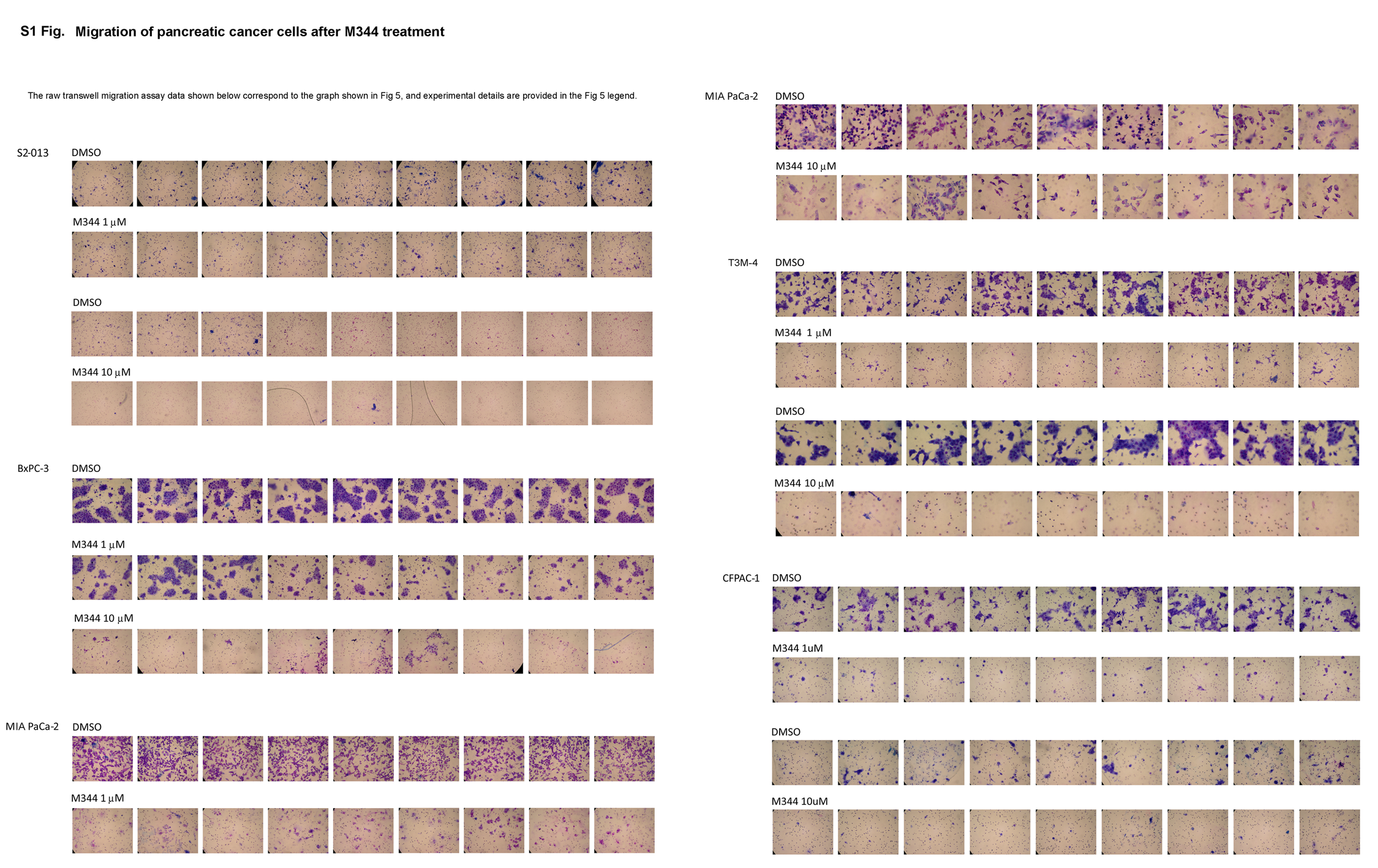

Supplement: S1 Fig — These transwell migration assay data correspond to the graph that is displayed in Fig 5, and the experimental details are listed in the legend for Fig 5. (TIF) [file pone.0273518.s001.tif]

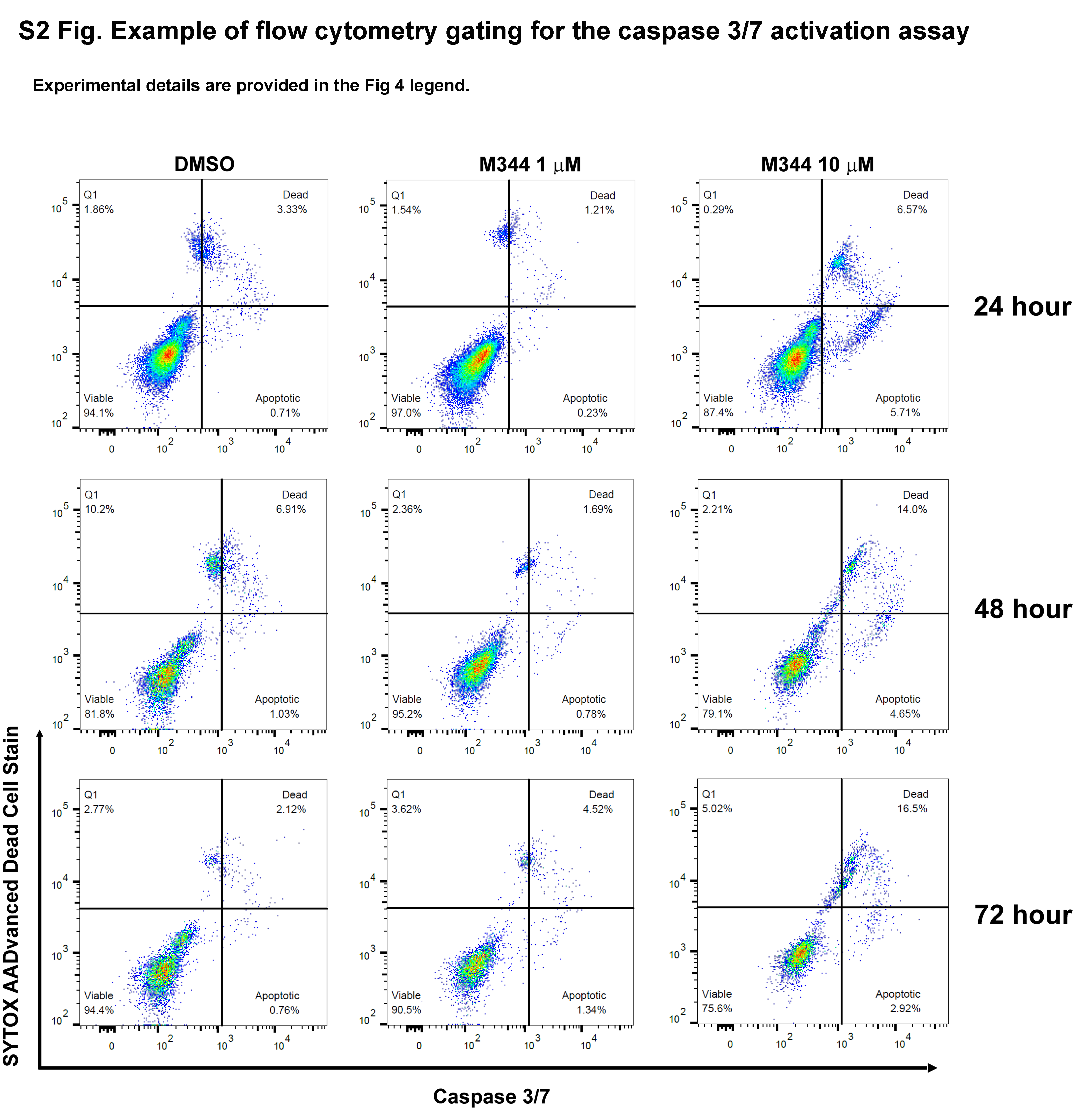

Supplement: S2 Fig — Details of the experimental process can be found in the legend for Fig 4. (TIF) [file pone.0273518.s002.tif]

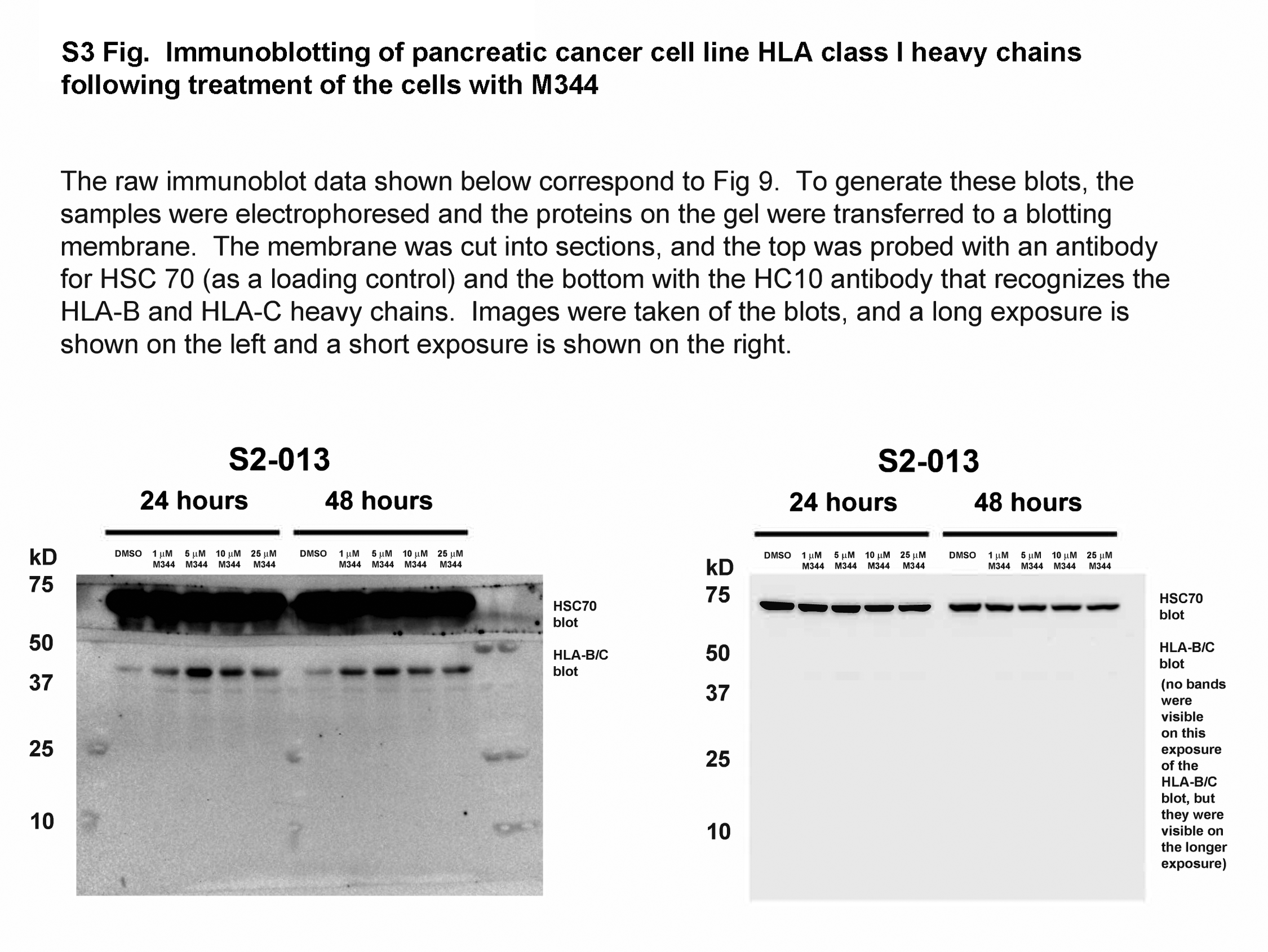

Supplement: S3 Fig — The immunoblot data displayed here correspond to Fig 9. The proteins were transferred after electrophoresis to a membrane that was then divided. The top portion was probed with anti-HSC 70 (loading control) antibody and the bottom portion was probed with the HC10 antibody (for HLA-B and–C heavy chains). The blots were imaged, and a long exposure and a short exposure are shown (on the left and right, respectively). (TIF) [file pone.0273518.s003.tif]
